# Tuberculosis in 0–5-year-old children following TB contact investigations: a retrospective study in a low burden setting

**DOI:** 10.3389/fped.2023.1145191

**Published:** 2023-06-19

**Authors:** Cassandre Pasqualini, Laure Cohen, Enora Le Roux, Marion Caseris, Albert Faye

**Affiliations:** ^1^ Assistance Publique des Hôpitaux de Paris, Service de Pédiatrie Générale et Maladies Infectieuses, Hôpital Robert Debré, Paris, France; ^2^ AP-HP, Nord-Université Paris Cité, Hôpital Universitaire Robert Debré, Unité d'Épidémiologie Clinique, Inserm, Paris, France; ^3^ ECEVE, Inserm, Paris, France; ^4^ Université Paris Cité, Paris, France

**Keywords:** tuberculosis infection, children, type of contact, risk factor, tuberculosis prophylaxis

## Abstract

**Introduction:**

We assessed the risk of tuberculosis (TB), the management and the outcomes of 0–5-year-old children after TB contact investigations in a low-burden setting.

**Method:**

All 0–5-year-old children who attended the TB clinic of Robert Debre Hospital, Paris, France, for a TB contact investigation between June 2016 and December 2019 were included in this retrospective study. The risk factors for TB were assessed using univariate and multivariate analyses.

**Results:**

A total of 261 children were included. Forty-six (18%) had TB, including 37 latent tuberculosis infections (LTBIs) and 9 active TB diseases. The prevalence of TB was 21% among high-risk contacts, i.e., household or close contacts and regular or casual contacts. There was no TB among intermediate- or low-risk contacts (0/42). Living under the same roof with (OR: 19.8; 95% CI: 2.6–153), the BCG vaccine (OR: 3.2; 95% CI: 1.2–8.3), contact duration >40 h (OR: 7.6; 95% CI: 2.3–25.3) and sleeping in the room of the index case (OR: 3.9; 95% CI: 1.3–11.7) were independently associated with TB. The BCG vaccine was no longer associated when the analysis was restricted to interferon gamma release assay results. Among children without initial LTBI, antibiotic prophylaxis was not prescribed for 2–5-year-old children or for 32/36 (89%) of 0–2-year-old children who had intermediate- or low-risk contact. Overall, none of these children experienced TB.

**Conclusion:**

In our low prevalence setting, the risk of TB in 0–5-year-old children following a household or close contact was high. Further studies are needed to better assess prophylaxis recommendations in intermediate or low risk contact.

## Introduction

Tuberculosis (TB) is one of the main infectious diseases responsible for childhood morbidity and mortality worldwide (1). France is a low-burden country with a rate of fewer than ten cases per 100,000 population diagnosed in the Paris area (2).

Not every child exposed to an index case of TB will develop a latent TB infection (LTBI) or active TB disease. The risk is related to many factors, such as the contagiousness of the index case, the proximity to the child, and the environmental conditions of the exposure (2–5). However, studies describing this risk among children are heterogeneous and from various settings, and no recent studies from low-burden countries address this issue.

Since 2012, the World Health Organization (WHO) has recommended that all 0–5-year-old children in household contact with an individual infected with bacteriologically confirmed susceptible pulmonary TB should receive TB prophylaxis (6).

For children between 0 and 5 years of age who encounter an index case, French guidelines recommend screening within 2 weeks of the last contact. Similar to other low-burden countries, anti-TB prophylaxis should be initiated for all 0–2-year-old children and 2–5-year-old children with LTBI (7–9).

However, in routine practice, antituberculosis prophylaxis for several weeks is not easy to manage for families, especially families of very young children. Moreover, such prophylaxis may lead to adverse events, which have been estimated to occur in up to 11% of cases (10). Thus, the risk-benefit ratio of systematic treatment for children younger than 2 years of age with low- or intermediate-risk TB contact is somewhat controversial.

The main objective of this study was to assess the prevalence of TB following TB contact investigations among 0–5-year-old children in a French low-burden setting. The secondary objectives were to assess the risk factors for TB and to describe the prophylactic management and outcomes of the children after the first screening.

## Population and methods

### Study design and population

This retrospective observational cohort study was carried out between June 1, 2016 and December 31, 2019 at the outpatient TB clinic of Robert Debré University Hospital, Paris, France.

All 0–5-year-old children who attended a specific TB clinic after contact with a person with bacillary pulmonary TB and with at least an 8-week follow-up were included.

### Definition

TB was defined as a condition resulting from the multiplication of TB bacilli (*M. tuberculosis*). LTBI was defined, according to French national guidelines, as a positive tuberculin skin test (TST) with a >10-mm induration in case of no previous BCG vaccination or a >15-mm induration in case of previous BCG vaccination or as a positive interferon gamma release assay (IGRA) result without any clinical or radiological signs of disease (8, 9).

Active TB disease was defined as clinical signs (i.e., general manifestations including weight loss, fatigue, fever, or symptoms related to pulmonary or extrapulmonary TB), radiological signs (i.e., parenchymal opacity, nodules, cavitary TB, miliary TB) and/or involvement of organs other than the lungs (i.e., pleura, mediastinal or peripheral lymph nodes, abdomen, genitourinary tract, skin, joints and bones, meninges) due to a multiplication of the bacilli that were identified through PCR and/or culture, or any combination of these.

Three types of contacts were categorized based on French public health authorities and WHO definitions (6, 8):

• High risk of TB contact: Household or close contact (a child living under the same roof, people sharing limited space daily) or regular or casual contact (contact with the index case, but for a shorter period with a cumulative contact time of more than 8 h if a sputum smear was positive or more than 40 h if a sputum smear was negative and a culture was positive).
• Intermediate risk of TB contact: Contact with sputum smear-positive individuals with a cumulative contact time of fewer than 8 h or contact with sputum smear-negative individuals and culture-positive individuals with a cumulative contact time between 8 and 40 h.
• Low risk of TB contact: Contact with sputum smear-negative and culture-positive individuals with pulmonary involvement but without cavitary TB and with a cumulative contact time of fewer than 8 h and not sharing the same home.

### Screening procedure

All 0–5-year-old children who had proven contact with an individual affected by pulmonary TB had to have been seen within the first 2 weeks following the diagnosis of the index case for a clinical examination, chest x-ray, and immunological test (TST and/or IGRA). Without initial evidence of TB infection, a second evaluation was scheduled within 3 months of the last potentially infectious contact. During this interval, antibiotic prophylaxis with isoniazid and rifampin was prescribed for 3 months for all 0–2-year-old children who had high-risk contact regardless of whether they had an LTBI and for all 2–5-year-old children with an LTBI. For children younger than 2 years of age who had a low or intermediate risk of TB, the physician was responsible for deciding whether to initiate treatment. In the case of LTBI, isoniazid and rifampicin were prescribed for 3 months. A third clinical and radiological screening was performed between 6 and 12 months after the first screening.

### Data collection

The data were collected from the medical records of the TB clinic. The contact and index case demographic and history characteristics, exposure time, type of contact, date and results of the first, second, and third screening tests and medical treatments were collected.

### Statistical analysis

For descriptive analysis, continuous variables are presented using the medians and first and third quartiles (*Q*1–*Q*3) and categorical variables using numbers (percentages). Potential TB-related factors were first investigated using univariate tests (*χ*^2^). The studied variables were the contact case's characteristics, the index case's characteristics, the exposure intensity, and the different types of contact according to the definitions given above. Then, a multivariate logistic regression model was developed and included as explanatory variables those whose *p* value was less than 0.2 in the univariate models and those known to be associated with TB in the literature. Some of these selected variables for the regression model were collinear and could not be used as explanatory variables within a single model; several multivariate models were therefore tested. Analytical statistic results are expressed by crude and adjusted odds ratios (ORs) and their 95% confidence intervals (95% CIs) and *p* values (two-tailed tests, *p* < 0.05 considered statistically significant).

A sensitivity analysis was carried out to study the robustness of the results according to the definition variation of the variable to be explained (TB). The previously mentioned tests were reapplied using a more sensitive definition of the TB event, considered positive for all patients with at least one positive IGRA during one of their three screenings among those with an IGRA. This analysis was carried out to study the potential impact of BCG vaccination on the relationship between TB and preidentified risk factors. Analyses were conducted using SAS® software (version 9.4).

### Ethical considerations

Data collection was approved by the French National Data Protection Commission (CNIL, number 20200526125843), and the local institutional review board approved this study (CEERD 2020-502).

## Results

### General characteristics of the study population

**Figure 1:**
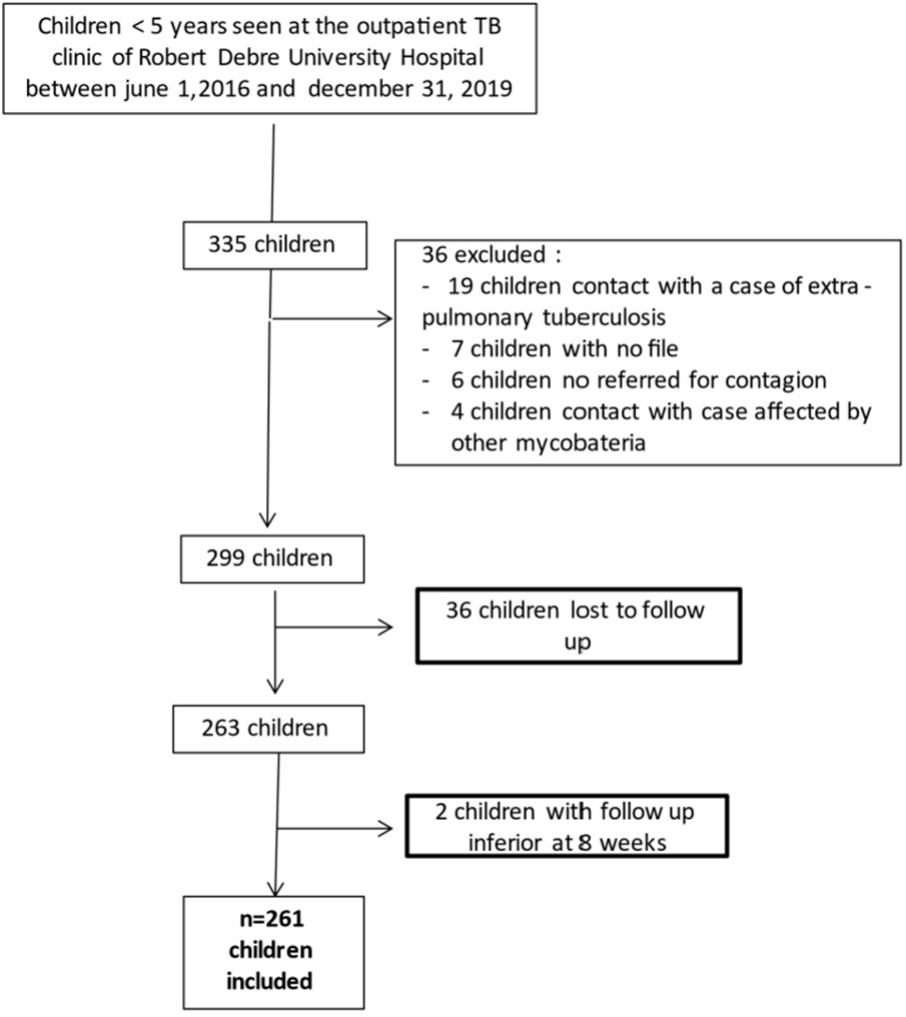
Flow chart.

Between June 1, 2016 and December 31, 2019, 335 children attended the Robert Debre Hospital TB clinic. Ultimately, 261 0–5-year-old children were included in this study (Figure 1). The children's characteristics are presented in Table 1.

**Table 1:** Demographic and baseline characteristics and type of TB cases contact of the population of the study.

	Total *N* = 261	
	*n*	%
Country of birth		
Metropolitan France	254	97.3
Other	7	2.7
Sex		
Female	139	53.3
Male	122	46.7
Comorbidity		
Yes	4	1.6
No	257	98.4
Geographical origin of the parents		
Metropolitan France	80	30.6
Sub-Saharan Africa	99	37.9
North Africa	37	14.2
Others	45	17.2
Age at first visit in TB clinic		
≤1 year	91	34.8
1–2 years	90	34.5
2–5 years	80	30.7
BCG vaccination		
Yes	175	67.7
No	84	32.4
History of tuberculosis in the family		
Yes	222	89.5
No	26	10.5
Type of contact		
Low risk	3	1.2
Intermediate risk	39	14.9
High risk		
Regular or casual contact	77	29.5
Household or close contact	142	54.4

There were no significant differences between the demographic characteristics of children aged 0–2 years and children aged 2–5 years, except for BCG vaccination, as children aged 0–2 years were less frequently vaccinated (60% vs. 82%) (Table 2).

**Table 2:** Frequency of TB in the children of the study according to their age (<2 vs. 2–5), baseline characteristics and type of TB cases contact.

	Under 2 years old (*N* = 181)		2–5 year old (*N* = 80)	
	*N* of patients (%)	*N* of TB (%) (*n* = 25)	*N* of patients (%)	*N* of TB (%) (*n* = 21)
Sex				
Male	87 (48)	12 (14)	35 (44)	7 (20)
Female	94 (52)	13 (14)	45 (56)	14 (31)
Missing data	0	0	0	0
Country of birth				
France	176 (97)	23 (13)	78 (97)	20 (26)
Other	5 (3)	2 (40)	2 (3)	1 (50)
Missing data	0	0	0	0
BCG vaccination				
No	71 (39)	6 (8)	13 (16)	1 (8)
Yes	109 (60)	19 (17)	66 (82)	20 (30)
Missing data	1	0	1	0
History of Tuberculosis				
No	151 (83)	22 (15)	71 (89)	19 (27)
Yes	18 (10)	2 (11)	8 (10)	2 (25)
Missing data	0	1	1	0
Type of contact				
Low or intermediate risk	32 (18)	0 (0)	10 (12)	0 (0)
High risk				
Regular or casual contact	62 (34)	6 (10)	15 (19)	0 (0)
Household or close contact	87 (26)	19 (22)	55 (69)	21 (38)
Missing data	0	0	0	0

184 children were tested with TST and IGRA, 70 with TST only and 7 with IGRA only. Of the 70 children who were screened by TST only, 64 were vaccinated (Supplementary Table S1).

Fifty-four percent of the children (142/261) had high-risk household or close contact, 29.5% (77/261) had a high risk related to regular or casual contact, 14.5% (39/261) had an intermediate risk, and 1.7% (3/261) had a low risk. Most (82%, 149/181) children less than 2 years of age had high-risk contact and 18% (32/181) low- or intermediate-risk contact, while 87% (70/80) of children between 2 and 5 years of age had high-risk contact and 12% (10/80) had low- or intermediate-risk contact (Table 2).

The median time from the index case diagnosis to the first TB consultation was 4 weeks [*Q*1–*Q*3: 3.0–7.9], and the time between the first and second screenings was 14 weeks [*Q*1–*Q*3: 10.4–15.6].

### Prevalence of tuberculosis infection

Among the 261 children, 46 (18%) had TB. Of these, nine (3%) had active TB disease, and 37 (14.5%) had LTBI.

Thirty-five children were diagnosed at the first screening and eleven at the second follow-up screening. All patients with active TB were asymptomatic or paucisymptomatic and diagnosed at the first screening. Five TB cases were mediastinal, three were pulmonary with negative culture, and one was miliary. All 11 children diagnosed at the second screening were over 2 years of age and had a high-risk household or close contact.

The prevalence rates of TB were 14% (25/180) among children aged 0–2 years and 27% (21/80) among children aged 2–5 years (*p* = 0.02). The prevalence rates of LTBI and active TB among children aged 2–5 years were 12% (22/181) and 2% (3/180) among children aged 0–2 years and 19% (15/80) and 7% (6/80) among children aged 2–5 years, respectively.

The median time between TB diagnosis of the index case and the diagnosis of TB (LTBI or active TB disease) was 5.7 weeks [IQR: 3.3–14.6]. This value was 5.7 weeks [IQR: 3.6–16] for children under 2 years old and 5.7 weeks [IQR: 2.8–14.5] for 2–5-year-old children.

### Risk factors for tuberculosis infection

The risk factors for TB after TB contact are described in Table 3. The prevalence of TB was 21% in high-risk contacts: 28% for high-risk contacts with household contact (40/142) and 8% for high-risk contacts with regular or casual contact (6/77); 0% in intermediate-risk contacts (0/39) and 0% in low-risk contacts (0/3). All the children with active TB disease had high-risk contact with the index case.

**Table 3:** Risk factor of TB in the children of the study according to the demographic and baseline characteristics and the type of TB cases contact.

	Nb of patient	Nb (%) of TB	Univariate analysis		Multivariate analysis			
					Model 1		Model 2	
	*n* = 261	*n* = 46	OR [IC_95%_]	*p*	aOR [IC_95%_]	*p*	aOR	*p*
Patient variables								
Sex								
Male	122	19 (16)	1	0.52	–	–		
Female	139	27 (19)	1.2 [0.6–2.3]					
Age at first visit in TB clinic								
<2 ans	181	25 (14)	1	0.02	1	0.49	1	0.31
2–5 ans	80	21 (26)	2.1 [1.1–4.1]		1.3 [0.6–2.7]		1.5 [0.7–3.4]	
Country of birth								
France	254	43 (17)	1	0.10				
Other	7	3 (42)	3.6 [0.8–16.5]					
BCG vaccination								
No	84	7 (8)	1	<0.01	1	0.01	1	0.02
Yes	175	39 (22)	3.2 [1.4–7.6]		3.1 [1.2–7.6]		3.2 [1.2–8.2]	
Missing data	2							
History of tuberculosis								
No	222	41 (18)	1	0.66				
Yes	26	4 (15)	0.8 [0.2–2.4]					
Missing data	13							
Index case variables								
Type of contact								
Low or intermediate risk	42	0 (0)	1	<0.01	1	<0.001		
High risk								
Regular/casual contact	77	6 (8)	3.4 [0.4–29.8]		3.6 [0.4–31.3]			
Close contact (household contact)	142	40 (28)	16.1 [2.1–120.8]		15.9 [2.1–121.1]			
Not living under the same roof	57	12 (21)	10.9 [1.4–87.8]		11.0 [1.4–89.5]			
Living under the same roof	85	28 (32)	20.1 [2.6–154.1]		19.8 [2.6–153.0]			
Relationship with the index case								
Parents and sibling	66	21 (31)	3.1 [1.2–8.1]	0.009			0.4 [0.1–1.5]	0.40
Other first degree family	74	9 (12)	0.9 [0.3–2.7]				0.4 [0.1–1.3]	
Collectivity	66	8 (12)	0.9 [0.3–2.8]				0.4 [0.1–1.4]	
Others	55	6 (11)	1				1	
Contact time								
<40 h	107	4 (4)	1	<0.01			1	0.001
>40 h	154	42 (27)	7.6 [2.9–20.1]				7.6 [2.3–25.3]	
Index case living under the same roof								
No	152	17 (11)	1	0.003				
Yes	109	29 (27)	2.7 [1.4–5.2]					
Index case sleeping in the same room								
No	218	28 (13)	1	<0.01			1	0.01
Yes	43	18 (41)	4.7 [2.3–9.6]				3.9 [1.3–11.7]	
Index case sleeping on the same bed								
No	237	34 (14)	1	<0.001				
Yes	24	12 (50)	5.8[2.4–13.9]					
Sputum smear positive								
Yes	145	28 (19)	1.4 [0.7–2.7]	0.31				
No	116	17 (15)	1					
Index case imaging								
Cavern	134	26 (19)	1.8 [0.8–4.2]	0.16			1.8 [0.6–5.1]	0.25
Lung nodule	49	11 (22)	2.5 [1.0–6.4]				2.2 [0.9–5.6]	
Other	78	9 (11)	1				1	

In the univariate analysis, age between 2 and 5 years, BCG vaccination, household or close contacts, relationship with the contact case, contact time more than 40 h, and index case living under the same roof, in the same room, and/or in the same bed were associated with TB.

Except for the 2–5-year-old age range, all these factors were associated with TB in multivariate logistic regression analysis (Table 3).

We performed a sensitivity analysis including only 184/261 children with an IGRA. Demographic characteristics and type of contact were similar in the children who had an IGRA and those who did not (Supplementary Table S2). The prevalence of TB was 12% (22/184). In this analysis, BCG was no longer significantly associated with TB (OR: 2.3; 95% CI: 0.7–7; *p* = 0.15) (Table 4).

**Table 4:** Risk factor of TB in the children of the study considering only the IGRA result according to the demographic and baseline characteristics and the type of TB cases contact.

	Nb of patient	Nb (%) of TB	Univariate analysis	
	*n* = 184	*n* = 22	OR [IC_95%_]	*p*
Patients variable				
Sex				
Male	79	8 (10)	1	0.7
Female	105	14 (13)	1.2 [0.5–2.9]	
Age at first visit in TB clinic				
<2 ans	124	9 (7)	1	0.01
2–5 ans	60	13 (22)	3.1 [1.3–7.7]	
Country of birth				
France	179	20 (11)	1	0.09
Other	5	2 (40)	5.0 [0.8–31.8]	
BCG vaccination				
No	56	4 (7)	1	0.15
Yes	128	18 (23)	2.3 [0.7–7.0]	
History of tuberculosis				
No	157	19 (12)	1	0.90
Yes	22	3 (14)	1.1 [0.3–4.0]	
Missing data	5			
Index case variables				
Type of contact				
Low or intermediate risk	37	0 (0)	1	0.009
High risk				
Regular or casual contact	44	1 (2)	0.8 [0.1–13.8]	
Household or close contact	103	21 (20)	9.2 [1.2–71.2]	
Relationship with the index case				
Parents and sibling	56	12 (21)	3.0 [0.8–11.5]	0.07
Other first degree family	68	4 (6)	0.7 [0.1–3.2]	
Collectivity	21	2 (9)	1.1 [0.2–7.5]	
Others	36	2 (5)	1	
*Missing data*	3			
Contact time				
<40 h	74	1 (1)	1	0.005
>40 h	110	21 (19)	8.5 [1.9–37.4]	
Index case living under the same roof				
No	85	17 (20)	1	0.002
Yes	99	5 (5)	5.0 [1.8–14.3]	
Sputum smear positive				
Yes	110	15 (14)	1.9 [0.7–5.2]	0.20
No	74	6 (8)	1	
Index case imaging				
Cavern	85	12 (14)	1.7 [0.5–5.0]	0.63
Lung nodule	43	5 (12)	1.6 [0.5–5.8]	
Other	56	5 (9)	1	

### Follow-up of the children and prophylaxis

Apart from the nine TB cases diagnosed at the first screening, no child presented active TB disease during the follow-up. Overall median follow up was 10 months and 3 days, ranging for 2–12 months.

All children with LTBI or less than 2 years old with high-risk contact received prophylactic antibiotic treatment with isoniazid and rifampicin. Among 0–2-year-old children with low or intermediate risk, only six out of 32 (19%) received antibiotic prophylaxis. None of the 26 children who did not receive prophylaxis subsequently presented with TB. None of the 55 2–5-year-old children with a high-risk household or close contact and no LTBI at the first screening received any antibiotic prophylaxis or were affected by active TB disease, but 11 (50%) had an LTBI diagnosed at the second screening. None of the 25 children with high-risk regular or casual contact or low- or intermediate-risk contact received any antibiotic prophylaxis, and none of them developed active TB disease.

## Discussion

In countries with a low incidence of TB, no study has described the management of children younger than 5 years of age after contact with an index case of TB in routine practice.

The prevalence of TB was 18%, with 14.5% LTBI and 3% active TB disease. These rates are similar to the 16.3% LTBI and the 4.7% active TB disease observed by Fox et al. in their meta-analysis, which included studies from high-income countries (5). In multiple studies, it has been reported that most children have a high risk of exposure to TB (5). However, the definitions of household or close contact vary considerably between different studies ranging from a broad definition including any known exposure to precise duration and/or proximity of exposure to define a high-risk exposure (11). Here, we used a combined definition using stringent criteria based on WHO and French guidelines to allow a more accurate assessment.

Children under five had a higher risk of developing TB infection after exposure than older children or adults (5, 12). In our study, the prevalence of TB infection was lower among children under 2 years (14%) than among children 2–5 years of age (26%). This difference might be explained by the type of exposure, as 69% of children between 2 and 5 years old had household contact compared to only 26% of children under two years old. Moreover, age between 2 and 5 years appeared to be a risk factor in the univariate analysis but not in the multivariate analysis.

Similar to other studies, it can be assumed that children between 2 and 5 years of age have the same risk of TB infection after exposure contact as children under 2 years of age (11, 13).

The prevalence of TB among children with high risk from a household or close contact was 28% vs. 8% for children with a high risk from a regular or casual contact. Many studies describe the prevalence of TB after household contact, with rates ranging from 22%–32% (14, 15) to 54% (16) or even 64%–68% (17, 18). These discrepancies might be related to the location of the study, i.e., a country with a low or high incidence of TB but also to the variation in definitions of household contact.

In our study, no children with low- or intermediate-risk contact contracted TB. To date, no studies have described the risk of TB for casual, low-risk, or intermediate contacts.

Consistent with the literature, we confirmed that children with household or close contact had a high risk (aOR: 16.1) of TB. Children with household contact living under the same roof were twice as likely to develop TB than children who did not live under the same roof as the person with TB (aOR: 19.8 vs. 11). Independently, having a contact time longer than 40 h and sleeping in the same room were also risk factors, with a related risk ranging from 2.1 to 5.2 (2, 4, 17).

Contrary to many studies (2–4, 16), the contagiousness of the index cases (smear sputum positivity and the presence of cavitary lesions) did not appear to be a risk factor for TB. The power of this study did not allow us to state a definitive conclusion.

The proportion of children vaccinated with BCG was high in our study, as in France, BCG vaccination is recommended for children living in the Paris region or born in a highly endemic country or with at least one parent originating from one of these countries. Surprisingly, BCG vaccination was associated with TB when the TST results of the screening were considered but not when the IGRA results were considered. It could be hypothesized that the TST overdiagnoses TB in vaccinated children compared to the IGRA. Therefore, our study is similar to others in that it confirmed the increased specificity of the IGRA for vaccinated children (19–22).

French national guidelines and others, such as the NICE guidelines from the United Kingdom, recommend systematic antibioprophylactic treatment for 0–2-year-old children regardless of the type of contact with an index case (7–9). Adherence to anti-TB chemoprophylaxis might be poor, and treatment might be associated with side effects (23–26). In our study, of the 32 children younger than 2 years who had intermediate- or low-risk contact, 26 (81%) did not receive prophylactic treatment, and none developed TB. The 11 children between 2 and 5 years of age who had low- or intermediate-risk contact also did not develop TB. This might suggest that if a precise assessment of the risk of exposure is available, and the exposure is low- or intermediate-risk exposure, the recommended systematic initiation of prophylaxis could eventually be discussed. However, the low number of children in our study prevented us from drawing any definitive conclusion, and this strategy was not adapted for low- or middle-income countries with a high TB incidence because of the higher risk of TB compared to low-incidence countries (6). We confirmed that for the children who had high-risk contact related to household or close contact, the initiation of prophylaxis was mandatory, as 20% of these children (11/55) without TB at the first screening were diagnosed with LTBI at the second screening visit (8, 9).

Our study had some limitations. Only three cases of low-risk contact with someone with TB were included, preventing us from discussing this type of exposure. There were also only a few cases of active TB disease, preventing a proper assessment of risk factors leading to this evolution. Moreover, children were not followed up for more than 1 year. Some children could have developed TB after the study period, even though we know that the incidence of new cases of TB among children under 5 years of age is the highest during the first year (5). Finally, this study included a small cohort from only one area, and the extrapolation of our results to all low-incidence countries should be performed with caution.

## Conclusion

Finally, among 0–5-year-old children following TB contact in a low-prevalence setting, we observed an 18% prevalence of TB, similar to that in previous international studies. We confirmed that among children, the risk of developing TB is closely associated with the type of contact, which should be assessed very precisely. As suggested in some studies, assessing TB with IGRAs rather than with TSTs seems accurate for children under 5 years of age, as BCG vaccination might lead to a false-positive TST result. In low-prevalence settings, prophylaxis for children under 5 years of age could be more closely adapted to a precise assessment of the type of contact. However, larger, prospective multicentric studies are strongly needed to better assess prophylaxis recommendations in these specific settings.

## What is known about this topic

- Tuberculosis is one of the main infectious causes of childhood morbidity and mortality around the world.
- The studies describing the risk of TB in under 5 years children following a contact with an index case are heterogeneous, from various settings and there are no recent studies from low burden countries addressing this issue.
- WHO recommended that all children under five, with household contact with a person with a bacteriologically confirmed pulmonary tuberculosis should receive tuberculosis prophylaxis.

## What the study adds

- We describe the risk to develop a TB infection after an exposure according to precisely defined types of contact with the index case.
- In countries with low TB incidence, the recommendation of systematic prophylaxis in children under five years exposed to TB case should be guided to a more accurate characterization of the type of contact.

## Data Availability

The original contributions presented in the study are included in the article/**Supplementary Material**, further inquiries can be directed to the corresponding author.
