# Supplementary material for: Tuberculosis in 0–5-year-old children following TB contact investigations: a retrospective study in a low burden setting
Source: Front Pediatr. 2023 Jun 19;11:1145191. doi: 10.3389/fped.2023.1145191 (PMC10315530; doi:10.3389/fped.2023.1145191)
Supplement: Supplementary file 1 [file Table1.pdf]

Supplementary Table 1 : Distribution of TST and IGRA's according to the age and BCG vaccine.

	TST + IGRA	TST	IGRA	Total
< 2 years	124	56	1	181
2-5 years	60	14	6	80
BCG vaccine	108	64	3	175

Supplementary Table 2: Description of the children with or without IGRA performed

	Children with IGRA (n=184)		Children whitout IGRA (n=77)	
	N	%	N	%
**Sex**				
Male	79	43	43	55
Female	105	57	34	45
**Age at first visit in TB clinic**				
<2 years	124	67	57	74
2-5 years	60	33	20	26
**Country of birth**				
France	179	97	75	97
Other	5	3	2	3
**BCG vaccination**				
No	56	30	28	36
Yes	128	70	49	64
**History of Tuberculosis**				
No	157	88	65	85
Yes	22	12	4	5
**Type of contact**				
Low and intermediate risk	37	20	5	6
High risk				
Regular or casual contact	44	24	33	43
Household or close contact	103	56	39	51
**Relationship with the index case**				
Parent and sibling	56	31	10	13
Other first-degree family	68	38	5	6
Collectivity	21	11	44	58
Other (neighbours, carer, second degree family)	36	20	18	23
**Contact time**				
<40h	74	40	33	43
>40h	110	60	44	57
**Index case living under the same roof**				
No	85	46	53	69
Yes	99	54	24	31
**Sputum smear positive**				
Yes	109	60	40	52
No	73	50	37	48
**Index case imaging**				
Cavern	85	46	49	64
Lung nodule	43	24	6	8
Other	56	30	22	28
